# Deep Sequencing of T-Cell Receptors for Monitoring Peripheral CD8^+^ T Cells in Chinese Advanced Non–Small-Cell Lung Cancer Patients Treated With the Anti–PD-L1 Antibody

**DOI:** 10.3389/fmolb.2021.679130

**Published:** 2021-07-09

**Authors:** Jin Sheng, Huadi Wang, Xiao Liu, Yunyun Deng, Yingying Yu, Pengfei Xu, Jiawei Shou, Hong Pan, Hongsen Li, Xiaoyun Zhou, Weidong Han, Tao Sun, Hongming Pan, Yong Fang

**Affiliations:** ^1^ Department of Medical Oncology, School of Medicine, Sir Run Run Shaw Hospital, Zhejiang University, Hangzhou, China; ^2^ Laboratory of Cancer Biology, Institute of Clinical Science, School of Medicine, Sir Run Run Shaw Hospital, Zhejiang University, Hangzhou, China; ^3^ Department of Human Genetics, University of Chicago, Chicago, IL, United States; ^4^ Hangzhou ImmuQuad Biotechnologies, LLC, Hangzhou, China; ^5^ Zhejiang-California International Nano-Systems Institute, Zhejiang University, Hangzhou, China

**Keywords:** next-generation sequencing, liquid biopsy, T-cell receptor repertoires, non–small cell lung cancer, anti–PD-L1 immunotherapy

## Abstract

**Background:** Atezolizumab, a high-affinity engineered human anti–PD-L1 antibody, has produced a clinical benefit for patients with advanced non–small-cell lung cancer (NSCLC). However, associated with T-cell regulation, the immunomodulatory effect of PD-L1 blockade and its biomarker in peripheral immunity remains elusive.

**Methods:** In a prospective cohort with 12 Chinese advanced NSCLC patients who received atezolizumab 1,200 mg every 3 weeks as a second-line treatment, blood samples were obtained before and 6 weeks after atezolizumab initiation, and when disease progression was confirmed. Patients were classified into a response or progression group according to response evaluation criteria in solid tumors (RECIST) 1.1. Fresh peripheral blood mononuclear cells (PBMCs) from patients were stained with antihuman CD3, CD8, and PD-1 antibodies for flow cytometry analysis. T-cell receptor (TCR)-β chains of CD8^+^ T cells were analyzed by next-generation sequencing (NGS) at the deep level. Diversity, clonality, and similarity of TCR have been calculated before and after treatment in both groups.

**Results:** Clonal expansion with high PD-1 expression was detected in all patients’ peripheral CD8^+^ T cells before the treatment of atezolizumab. Unlike the progression group, the diversity of TCR repertoire and singletons in the TCRβ pool increased over time with atezolizumab administration, and the TCR repertoire dynamically changes in the response group. The percentage of CD8^+^ PD-1^high^ terminal exhausted T cells declined in the response group after the PD-L1 blockade. Two patterns of TCR changes among patients who received PD-L1–targeted immunotherapy were observed.

**Conclusions:** Deep sequencing of the T-cell receptors confirmed the existence of CD8^+^ PD-1high T cells with an exhaustion phenotype in Chinese NSCLC patients. Our study demonstrated that efficient anti–PD-L1 therapy could reshape the TCR repertoire for antitumor patients. Furthermore, singleton frequency may help us select patients who are sensitive to anti–PD-L1 immunotherapy.

## Introduction

Non–small cell lung cancer (NSCLC) that accounts for almost 85 percent of total lung cancer patients is still the leading cause of cancer-related mortality, especially in those with advanced disease (Sui et al., 2018; Seigel et al., 2017). Over the past few years, the introduction of the epidermal growth factor receptor (EGFR), tyrosine kinase inhibitors (TKIs), and anaplastic lymphoma kinase (ALK)-based target therapy dramatically improved the therapeutic efficacy for lung cancer patients with specific gene mutations (Califano et al., 2017; Hida et al., 2017; Peters et al., 2017; Kiura et al., 2018; Novello et al., 2018). However, tumor heterogeneity inevitably leads to resistance of targeted therapies from multiple mechanisms (Tsui et al., 2018). Encouragingly, immune checkpoint blockade (ICB) immunotherapy had changed the landscape for treating advanced NSCLC, with a more durable response than targeted therapy and chemotherapy.

Several inhibitory immunoreceptors, including but not limited to CTLA-4, PD-1, TIM3, and LAG3, have been studied in some solid tumors in the past decades. Moreover, the most successfully developed ICB therapy is anti–PD-1/PD-L1 therapy, since atezolizumab, nivolumab, and pembrolizumab have been approved for NSCLC (Ribas and Wolchok, 2018). The PD-1/PD-L1 signaling pathway is one of the self-protection mechanisms of tumors against the endogenous immune response (Taube et al., 2012). Conventionally, the primary PD-1 ligand expressed on the surface of tumor cells or antigen-presenting cells is PD-L1, with PD-1 on T cells to trigger inhibitory signaling (Curiel et al., 2003, 2007; Keir et al., 2008; Lin et al., 2018). T cells respond highly specific to particular antigens as a consequence so that the gene encoding their adaptive immune receptors is generated somatically through a process that creates unique sequences when they encounter the cognate antigen. The generation process of adaptive immune receptor genes results in a tremendously diverse repertoire of distinct T-cell receptors (TCR) to bind the particular antigens (Robins et al., 2010; Warren et al., 2013; Zarnitsyna et al., 2013; Kaplinsky and Arnaout, 2016; Briney et al., 2019). A somatic recombination process of the variable (V), diversity (D, beta chain), and joining (J) exons generates a high degree of TCR diversity and defines the highly variable complementary determining region 3 (CDR3) (Lee and Prins, 2016). CDR3 characterizes the antigen recognition specificity of each T-cell clone. Therefore, deep sequencing data of the T-cell antigen receptor repertoire give us access to a better understanding of T lymphocyte population dynamics in response to therapy (Cowell, 2020).

The application of TCR repertoire deep sequencing to reveal lymphocyte responses in cancer or immunotherapy is widespread (Kirsch et al., 2015; Schrama et al., 2017). The majority of studies have focused on the T-cell receptor beta locus. Studies of clonal richness and diversity of T lymphocyte population have ranged from tumor-infiltrating T cells and sentinel lymph node T cells to T lymphocytes in peripheral blood. Tumor-infiltrating T cells are the front line of identifying tumor antigens in the tumor microenvironment, resulting in considerable heterogeneity. In NSCLC, abundant TCR sequences have been classified as ubiquitous or regional. As for peripheral blood TCR repertoires, despite that studies found little overlap (from 19 to 25%) between peripheral blood and TIL TCR repertoires so far, evidence confirmed the peripheral TCR repertoire correlated with TILs, and their alteration reflects various aspects of the disease. Moreover, a study (Liu et al., 2019) suggested that peripheral TCR diversity decreases as the disease progresses in lung cancer.

The blockade of PD-1:PD-L1 interactions contains two options: anti–PD-1 and anti–PD-L1. PD-1 is expressed by activated T cells that bind to PD-L1 in the tumor microenvironment. However, during chronic exposure of tumor antigen, T cells undergo exhaustion with upregulation of inhibitory checkpoints, typically PD-1, as one of the tumor escape mechanisms. Blocking of PD-1 is expected to change these exhausted T cells’ behavior directly and hopefully reinvigorate them. In contrast, the blockade of PD-L1 produces more possibilities due to broad cell types expressing PD-L1 and the additional ligand B7-1. Intriguingly, the study from Fridman et al. (2017) has given us crucial evidence that anti–PD-L1 treatment during chronic lymphocytic choriomeningitis virus (LCMV) infection induced the interleukin-7 receptor (IL-7R or CD127) on exhausted T cells. IL-7 acts as a pivotal factor for the generation of memory T-cell phenotype (Schluns et al., 2000) and down regulates PD-1 expression on the CD8^+^ T cells during chronic LCMV infection. To confirm if similar regulation happens in cancer anti–PD-L1 immunotherapy, monitoring PD-1 expression in peripheral blood could give us a preliminary insight into the invigoration of T-cell exhaustion of anti–PD-L1 in cancer patients.

Collectively, in the present prospective cohort study, the expression of PD-1 and TCR repertoire was analyzed through high-throughput sequencing in blood samples from Chinese advanced NSCLC patients who had received anti–PD-L1 checkpoint blockade immunotherapy, aiming at exploring predictive and monitoring biomarkers before treatment and selecting patients to exhibit better clinical outcomes.

## Materials and Methods

### Study Design and Cohort

We performed a prospective cohort study of patients with metastatic lung cancer treated with atezolizumab (MPDL3280A), a PD-L1 inhibitor, at Sir Run Run Shaw Hospital, Zhejiang University. This study was approved by the Institutional Review Board (IRB) of the hospital. All patients received atezolizumab 1,200 mg intravenously every 3 weeks. Critical inclusion criteria for this study cohort were cytologically confirmed stage IIIB/IV squamous carcinoma or adenocarcinoma of NSCLC, Eastern Cooperative Oncology Group performance status (ECOG-PS) of 0–1, 18 years old or older measurable lesion confirmed by response evaluation criteria in solid tumors (RECIST) 1.1, and disease recurrence after one prior platinum-containing regimen. Patients with a known autoimmune disease, symptomatic interstitial lung disease, systemic immunosuppression, or prior immune-based therapies were excluded. Smoking status was classified as smokers and nonsmokers. Smokers have been referred to as those patients who smoke more than ten cigarettes per day and with cigarette withdrawal less than 2 years.

### Therapeutic Efficacy Analysis in Response to Atezolizumab

Tumor responses to atezolizumab were evaluated by a CT scan every 6 months. Clinical responses such as complete response (CR), partial response (PR), stable disease (SD), and progressive disease (PD) were determined based on RECIST 1.1. Patients were further categorized into the response group based on tumor shrinkage, while the progression group included patients who achieved PD (Table 1). The median follow-up was 5.0 (IQR 2.75–7.35) months at the endpoint. The median PFS was 4.4 (95% CI: 2.9–5.9) months among all patients.

$$f\left(x\right)=a_{0}+\sum_{n=1}^{\infty }\left(a_{n}\cos\frac{n\pi x}{L}+b_{n}\sin\frac{n\pi x}{L}\right).$$

**TABLE 1:** Demographic and clinical characteristics of patients.

Patient ID	Gender	Age	Histology	Smoking history	Cycles	Best response	Efficacy group	PFS/months
1	Male	61	SCC	Previous smoker	3	PD	Progression	2.9
2	Male	63	SCC	Previous smoker	4	PD	Progression	2.7
3	Male	59	SCC	Smoker	2	PD	Progression	1.4
4	Male	51	SCC	Previous smoker	11	PR	Response	7.7
5	Male	58	ADN	Previous smoker	8	PD	Progression	5.6
6	Male	52	ADN	Smoker	9	SD	Response	6.3
7	Male	75	SCC	Smoker	6	PD	Progression	4.3
8	Male	45	SCC	Smoker	12	SD	Response	8.3
9	Male	49	SCC	Smoker	6	SD	Response	4.4
10	Male	73	ADN	Previous smoker	3	PD	Progression	2.1
11	Female	58	SCC	Nonsmoker	13	SD	Response	9.3
12	Female	60	ADN	Nonsmoker	8	SD	Response	5.6

SCC refers to squamous carcinoma; ADN refers to adenocarcinoma.

PD: progressive disease; SD: stable disease; PR: partial response.

### PBMC Preparation and CD8^+^ T-Cell Isolation

We collected 25 samples of peripheral blood mononuclear cells (PBMCs) from 12 Chinese advanced NSCLC patients. The samples were collected at baseline and 6 weeks after the initiation of treatment within a 3-day time window. PBMCs were immediately isolated from each sample using Ficoll-Hypaque 1077 (Sigma-Aldrich) gradient centrifugation. Total CD8^+^ T cells were isolated from PBMCs with the BD IMag^™^ Anti-Human CD8 Magnetic Particles kit (catalog no. 557766).

### Flow Cytometry

For the flow cytometry analysis, all antibodies were purchased from BioLegend (San Diego, CA). Cells were stained with antihuman CD3 (HIT3a), CD8 (SK1), and PD-1 (EH12.2H7), and analyzed on a BD LSRFortessa^TM^ flow cytometer (Becton Dickinson) using FlowJo vX.0.7 software. Gates were set on CD3^+^ cells with forward and side scatter, and doublets were gated out (Supplementary Figure S1).

### TCR-β Full-Length Amplification and Sequencing

RNA extraction of pure CD8^+^ T cells was performed following the RNeasy Plus Mini Kit (Qiagen). The samples were analyzed by high-throughput sequencing of TCR full length using the ImmuHub^®^ TCR profiling system at the deep level (ImmuQuad Biotech, Hangzhou China). Briefly, a 5′ RACE unbiased amplification protocol was used. Sequencing was performed on an Illumina MiSeq^®^ system with PE300 mode (Illumina), as previously described (Wang et al., 2019). A post-sequencing algorithm was applied to raw sequencing data for PCR and sequencing error correction and V, D, J, and C gene segments mapping with IMGT^®^. The resulting nucleotide and amino acid sequences of CDR3 of TCRβ were determined, and those with out-of-frame and stop codon sequences were removed from the identified TCR-β repertoire. We further defined each TCR-β clonotype’s amounts by adding the numbers of TCR-β clones sharing the same nucleotide sequence of CDR3. The TCR-β repertoire analysis algorithm was based on R (version 3.5.1). The total-sequencing reads and the successfully aligned reads are listed in Supplementary Table S1 in detail.

### Diversity, Clonality, and Similarity Calculation

*Pielou’s evenness index* (Pielou, 1966) was calculated to estimate the diversity of TCRβ repertoire, according to the equation:

$$\mathrm{Pielou}\;\mathrm{index=}\frac{\mathrm{Shannon}\;\mathrm{index}\;H}{\ln N}=\sum_{i=1}^{N}\frac{p_{i}\left(\ln\;p_{i}\right)}{\ln N},$$

Clonality index=1-Pielou index,

where *p*_*i*_ is the proportion of sequence, *i* is relative to the entire sequences, and *N* is the total quantity of clonotypes. The *Pielou index* describes the equability of a TCR repertoire and is positively related to the diversity represented by the *Shannon index*.

Singleton means one unique TCR clone, and the entire singletons in one patient TCR repertoire have shown a strong correlation with the percentage of naive T cells (Britanova et al., 2014), which are the backbone of immune repertoire diversity. *Singleton frequency*, which calculated the proportion of singletons in the total clonotypes, is an essential factor in estimating the total repertoire clonality. In this study, we illustrated *singleton frequency* in donut charts, where the inner layer includes the frequency of singleton (“1,” T-cell clones that only had one sequence read) and high-order (“2+,” T-cell clones that had more than two sequence reads) clonotypes. Repertoire clonality was depicted as the “2+” portion in the donut chart.

Last, to estimate the overlap of TCR-β clonotypes between time points or biological compartments, the *Baroni-Urbani and Buser overlap index* (BUB) (Zhang et al., 2017) was calculated according to the following formula:

$$BUB=\frac{n_{12}}{n_{1}+n_{2}-n_{12}},$$

where $n_{1}$ is the number of clonotypes present at time point 1, $n_{2}$ is the number of clonotypes present at time point 2, and $n_{12}$ is the number of clonotypes presenting in both time points.

### Statistical Analysis

The Student’s t-test (two-tailed) was adopted to compare the Pielou’s evenness index, singletons frequency, and BUB overlap index between data before and after treatment. Two-way ANOVA analyzed the differences between repeated-measures data during therapy. A *p*-value of 0.05 was considered statistically significant. The Student’s t-test and two-way ANOVA were calculated by GraphPad Prism version 6.0 (La Jolla, CA, United States). The donut chart was made by R (version 3.4.0). A survival analysis was conducted using both Kaplan–Meier log-rank analysis and Cox proportional hazard models.

## Results

### Patient Characteristics and Therapeutic Efficacy

**FIGURE 1:**
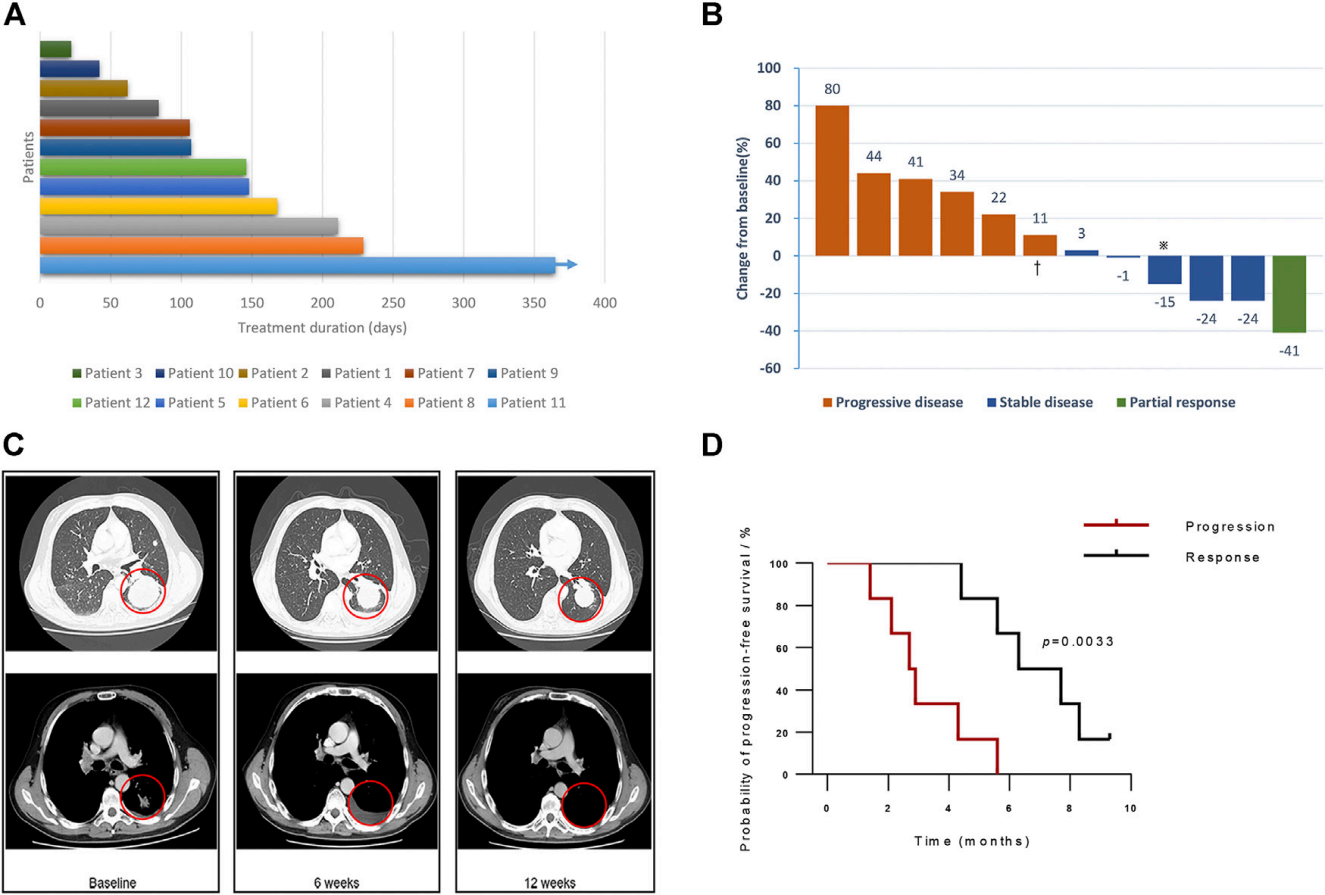
Clinical efficacy of PD-L1 blockade. **(A)** Investigator-assessed duration of treatment. The arrow indicates censored patient with follow-up ongoing after one year. **(B)** The confirmed maximum reduction of target lesions, according to the investigator and authorized radiologist. Colors of bars representing the best response confirmed during follow-ups. Red bars represent progressive disease, blue bars represent stable disease, and green bars show partial response. Patient two was confirmed of nontarget lesion progression during the one-year follow-up. **(C)** Representative computed tomography (CT) scans of Patient 11, who achieved a partial response to atezolizumab. **(D)** Comparison of estimated progression-free survival of the response group and the progression group (*p* < 0.005). Note: ※: Patient 11, †: Patient 2.

The primary characteristics of the 12 enrolled patients in this study cohort are summarized in Table 1. Briefly, eight patients had squamous carcinoma, and the remaining had adenocarcinoma. 83.3 percent of the cohort were current (*n* = 2) or former smokers (*n* = 8). All patients had an ECOG performance status of 1. The response group had a significantly younger average age than the progression group (53.5 vs. 64.8 years, *p* < 0.05, Supplementary Table S2). In this study, patients received a median of seven cycles of PD-L1 blockade immunotherapy, while half of them progressed after six cycles of treatment (Figure 1A). Among the response group, one patient (Patient 4) presented PR (Figure 1B), and five patients reached SD as their best response to the treatment during follow-ups (Table 1; Figure 1B). One patient (Patient 11) in the response group continued to respond to atezolizumab for more than 12 months, although the target lesions did not shrink remarkably as evaluated by computed tomography (Figure 1C). Comparing with the progression group (median 2.7 months, 95% CI: 1.7–3.7), the PFS significantly prolonged in the response group (median 6.3 months, 95% CI: 3.8–8.8; Kaplan–Meier estimate *p* < 0.005, Figure 1D).

### CD8^+^PD-1^high^ Terminal Exhausted T Cells Decreased in Response to PD-L1 Blockade

**FIGURE 2:**
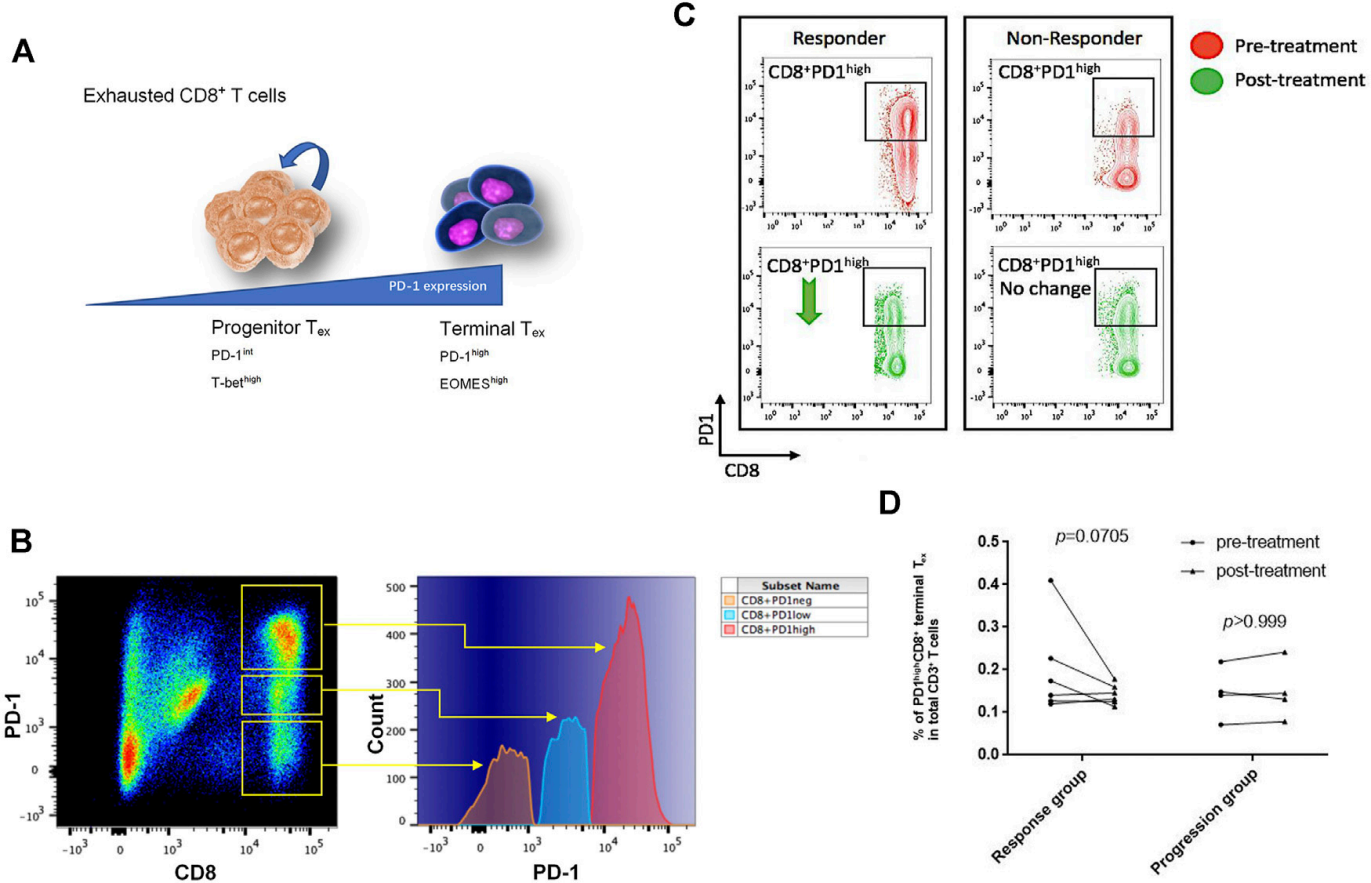
CD8^+^PD-1^high^ T cells decreased for responding to PD-L1 blockade treatment. **(A)** The CD8^+^PD-1^high^ subset was identified as terminal exhausted T cells, which highly expressed the EOMES transcription factor, while CD8^+^PD-1^int/low^ population that expressed the T-bet transcription factor was known as progenitor exhausted T cells (Wherry and Kurachi, 2015). **(B)** According to the log-density plot of the PD-1 expression level, three subsets of CD8^+^ T cells were found in Chinese non–small-cell lung cancer patients’ peripheral blood. There were PD-1^high^, PD-1^low^, and PD-1^neg^ CD8 T^+^ cells. **(C)** Two representatives illustrated that the percentage of CD8^+^PD-1^high^ T cells in the response group decreased, whereas it did not have an apparent change in the progression cohort after PD-L1 blockade treatment. **(D)** The percentage of CD8^+^PD-1^high^ T cells in each patient before and after treatment (*p* = 0.0705 in the response group; *p >* 0.999 in the progression group).

When tumor antigens persist in, exhausted T cells (T_ex_) form with the growing levels of persistent PD-1 expression, inducing a less functional state of exhaustion among cognate antigen-specific T cells. T_ex_ cells in the early stage retain the capability of renewal and are characterized by intermediate expression of PD-1. Early T_ex_ cells as a progenitor population give rise to terminal T_ex_ cells with high expression of PD-L1, which loses the ability of proliferation upon tumor antigen stimulation (Figure 2A) (McLane et al., 2019). Among the enrolled NSCLC patients, we confirmed PD-1–positive CD8^+^ T cells in peripheral blood and further classified them into PD-1^high^, PD-1^low^, and PD-1^neg^ subsets by the expression level of PD-1 (Figure 2B). The PD-1^high^ subsets were regarded as terminal T_ex_ cells.

The average number of sequencing reads was 851148 ± 120135 (range 69,600–1,892,801), and the average aligned reads were 638721 ± 84,218 (range 45,909–1,385,898). Details of the total sequencing reads and successfully aligned reads are listed in Supplementary Table S1. The number of CD8^+^PD-1^high^ terminal T_ex_ cells in the responders’ circulation decreased after anti–PD-L1 antibody treatment, whereas there was almost no change of this subset in the progression group (Figure 2C). After initiation of atezolizumab, in the response group, the percentage of PD-1^high^CD8^+^ terminal T_ex_ in total CD3^+^ T cells dropped from 12.5 ± 2.76 (95% CI: 5.38–19.6) to 6.38 ± 1.03 (95% CI: 3.73–9.02; *p* = 0.0705 posttreatment vs. pretreatment) after PD-L1 blockade treatment (Figure 2D). However, in the progression group, there was no significant difference in the percentage of PD-1^+^CD8^+^ T cells before and after treatment (Figure 2D; 8.86 ± 2.58 vs. 8.86 ± 2.84, *p* > 0.999).

### PD-L1 Blockade Changes TCR Repertoire Diversity

**FIGURE 3:**
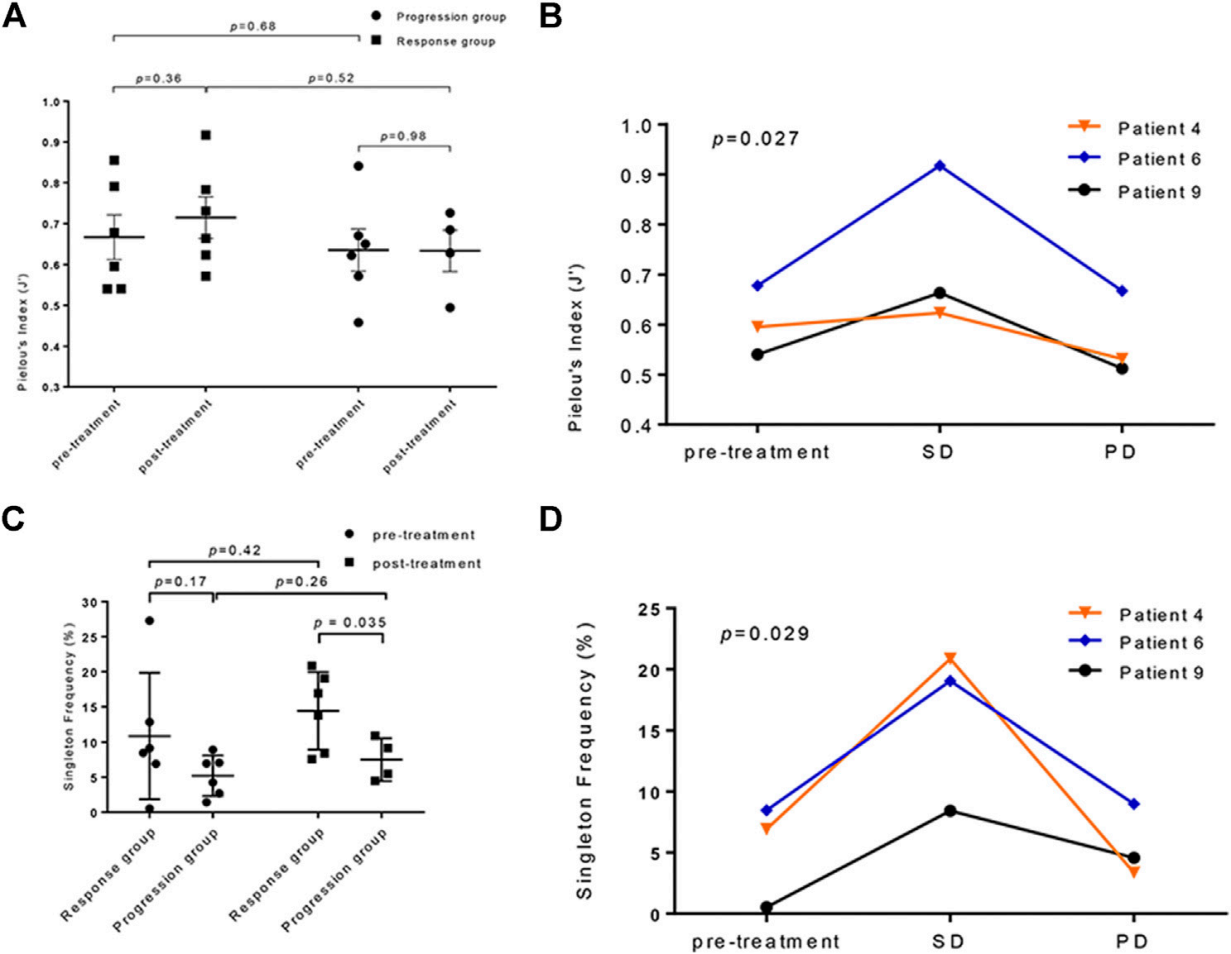
The analysis of the T-cell receptor (TCR) repertoires diversity in anti–PD-L1–treated patients. **(A)** The comparison of TCR repertoire evenness changes in the progression and response groups calculated by Pielou’s index (*p* = 0.36 in the response group; *p* = 0.98 in the progression group; response vs. progression: pretreatment *p* = 0.68, posttreatment *p* = 0.52). **(B)** The dynamic observation of TCR repertoire evenness at different disease statuses of three patients who responded to atezolizumab (*p* = 0.027). SD: stable disease; PD: progressive disease. **(C)** The singleton frequency in the response and progression groups before (*p* = 0.17) and after (*p* < 0.05) treatment. (pretreatment vs. posttreatment: *p* = 0.42 in the response group, *p* = 0.26 in the progression group). **(D)** The changes of the singleton frequency of the TCR repertoire in three patients responded to atezolizumab during treatment (*p* = 0.029).

For a given total number of distinct clonotypes in the repertoire, the maximally diverse repertoire consists of uniformly distributed adaptive immune receptors (*pi* are all equal in the formula) (Cowell, 2020). The evenness of the TCR repertoire represented by Pielou’s index increased in the response group after treatment (*p* = 0.36, paired *t*-test; Figure 3A); in contrast, those who did not respond to atezolizumab showed no change in the evenness of the TCR repertoire (*p* = 0.98, unpaired *t*-test). To assess the dynamic nature of the TCR repertoire diversity representing the whole course of effective anti–PD-L1 therapy, we collected blood samples from three patients in the response group with their consents when the progressive disease was confirmed. It is noticeable that the evenness of the TCR repertoire maintained or significantly elevated when the targeted lesions continued to respond to atezolizumab. However, Pielou’s index dramatically dropped when the disease progressed (*p* < 0.05, 2-way ANOVA; Figure 3B).

The changes in the singleton frequency reflect the clonal proliferation of the TCR repertoire during anti–PD-L1 immunotherapy. Before treatment, the singleton frequency between the response and the progression group showed no significant difference (10.86 ± 3.67% vs. 5.22 ± 1.77%; *p* = 0.17, unpaired *t*-test; Figure 3C). Nevertheless, after the initiation of atezolizumab, the gap between the mean singleton frequency of the response and progression group grew (14.44 ± 2.25% vs. 7.51 ± 1.52%; *p* < 0.05, Welch’s *t*-test; Figure 3C), which interpreted a better T-cell proliferative ability in patients who responded to PD-L1 blockade than in those who did not. Similarly, the singleton frequency of the same three patients in the response group was monitored during follow-ups. The clonal proliferation of the TCR repertoire dramatically elevated after the initiation of atezolizumab during stable disease. After progression, these three patients all had a decreased singleton frequency, indicating a lower level of richness of the TCR repertoire (*p* < 0.05, 2-way ANOVA; Figure 3D). There is not a clear correlation between the pretreatment singleton frequency and PFS (Supplementary Figure S2).

Also, we calculated the Baroni-Urbani and Buser index to measure the overlap among TCR-β clonotypes in the response and progression groups (Supplementary Figure S3). The average BUB index is moderately lower in the response group than that in the progression group (0.039 ± 0.0099 vs. 0.054 ± 0.017; *p* = 0.44, unpaired *t*-test), indicating the TCR similarity of patients in the response group is at a lower level than that of those in the progression group.

### TCR Repertoire Changes due to anti–PD-L1 Treatment Had Typical Patterns Featured by Top Clonotype and Singleton Frequency

**FIGURE 4:**
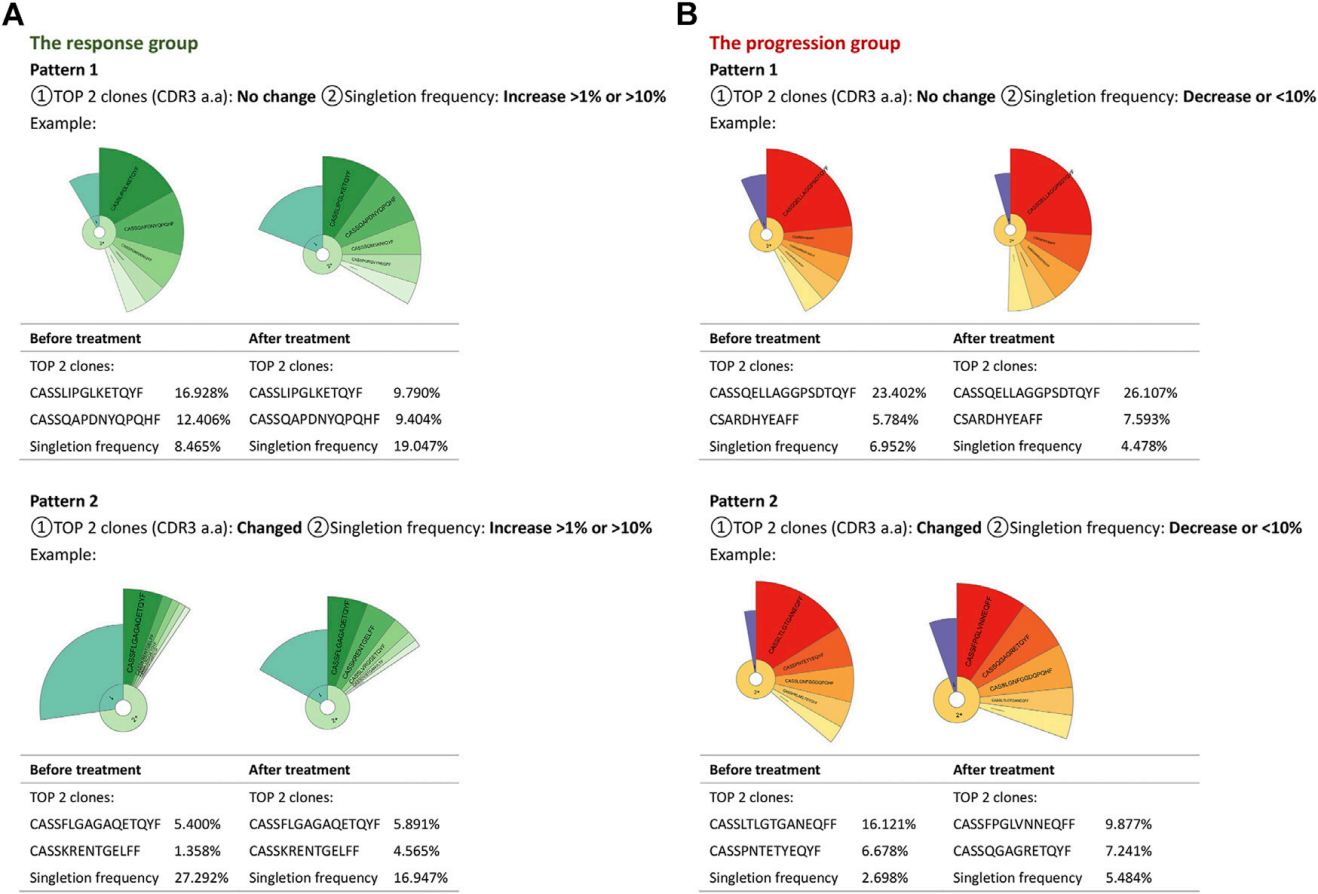
Changing patterns of the TCR repertoire in the progression and response groups. Donut charts and tables summarized two patterns in each group. Each pattern took one patient as an example in either group. The fan-shaped area depicts the corresponding clonal frequency. The area of “1” in the donut chart represented the frequency of singletons. “2+” area was the TCR sequences whose reads were more than two. A larger radian meant a higher frequency. The top five clones’ amino acid sequences of complementary determination region 3 (CDR3) of TCR were shown, and the frequencies of the top two clones were listed. The radian marked with the amino acid sequence displayed the frequency of the corresponding T-cell clones.

According to the clonotype and singleton frequency changes of the TCR repertoire before and after PD-L1 blockade immunotherapy, we summarized two patterns for every group, aiming at exploring a portable way to select proper patients for atezolizumab. In the first pattern of the response group, the singleton frequency of the TCR repertoire was increased primarily without variation of the top two clonotypes of CDR3 (Figure 4A). Pattern two had a decent percentage of singletons and was distinguished from the first pattern by the varied top two clones of the amino acid sequence of the CDR3 (Figure 4A). As for the progression group, pattern one had no change in neither singleton frequency nor top two clones of the TCR repertoire; in pattern two, the TCR repertoire of progressed patients maintained a small percentage of singletons in total clonotypes during treatment, but the top two clones changed (Figure 4B). The details of the top two clones of all patients before and after treatment are shown in Table 2. All information is given in Supplementary Table S3.

**TABLE 2:** The Top two clones’ amino acid sequences of complementary determination region 3 (CDR3) of TCR of each patient before and after treatment.

Group	Patient ID	Time point	Top 1 clone (CDR3)		Top 2 clone (CDR3)	
Progression	1	Pretreatment	11.80%	CASHRAGNEYEQYF	8.77%	CASSVAGTADYEQYF
		Posttreatment	NA	NA	NA	NA
	3	Pretreatment	43.09%	CASSFGTFGDGYT	4.45%	CASSLTSEHRFTDTQYF
		Posttreatment	NA	NA	NA	NA
	2	Pretreatment	16.12%	CASSLTLGTGANEQFF	6.68%	CASSPNTETYEQYF
		Posttreatment	9.88%	CASSFPGLVNNEQFF	7.24%	CASSQGAGRETQYF
	10	Pretreatment	8.45%	CASSEWGDTQYF	7.59%	CASRPSGTGGYNEQFF
		Posttreatment	9.04%	CASSEWGDTQYF	4.57%	CASRPSGTGGYNEQFF
	7	Pretreatment	23.40%	CASSQELLAGGPSDTQYF	5.78%	CSARDHYEAFF
		Posttreatment	26.11%	CASSQELLAGGPSDTQYF	7.59%	CSARDHYEAFF
	5	Pretreatment	9.33%	CASSEQGSGYEQYF	2.63%	CASSLNKGYGYTF
		Posttreatment	7.82%	CASSYSYEQYF	3.59%	CASSHPTGVEQYF
Response	9	Pretreatment	16.93%	CASSLIPGLKETQYF	12.41%	CASSQAPDNYQPQHF
		Posttreatment	9.79%	CASSLIPGLKETQYF	9.40%	CASSQAPDNYQPQHF
	12	Pretreatment	2.73%	CASSVKGSSGPLHF	2.08%	CATSSQDNTEAFF
		Posttreatment	9.94%	CASSVKGSSGPLHF	3.71%	CATSSQDNTEAFF
	6	Pretreatment	11.22%	CASSYSYEQYF	6.43%	CASSFGQGVYNEQFF
		Posttreatment	4.12%	CASRGTGLYNSPLHF	2.58%	CASSQPGQGTGELFF
	4	Pretreatment	19.50%	CSADGTSGNIQYF	14.21%	CASSQGQGGQPQHF
		Posttreatment	14.00%	CSADGTSGNIQYF	7.16%	CASSQGQGGQPQHF
	8	Pretreatment	5.40%	CASSFLGAGAQETQYF	1.36%	CASSKRENTGELFF
		Posttreatment	5.89%	CASSFLGAGAQETQYF	4.57%	CASSKRENTGELFF
	11	Pretreatment	14.07%	CATQWGQLALHF	12.58%	CATEDGRFRQYF
		Posttreatment	7.79%	CASSEGRGANGYTF	7.08%	CASSFTAGAETQYF

## Discussion

PD-L1 blockade immunotherapy has been proven to induce durable tumor remissions through T-cell response to cancer, and it remains unclear how these antibodies work in modulating T-cell immunity (Robert et al., 2014). Besides, serial monitoring of checkpoint inhibitors’ efficacy needs easily accessible tumor biomarkers. Therefore, the TCR repertoire has become a focus in several studies since checkpoint inhibitor blockade dramatically changed the treatment for advanced cancer patients. However, exploratory studies of peripheral TCR diversity analysis among advanced cancer patients treated with different checkpoint inhibitors blockade have reached conflicting conclusions. So, we did this pilot study to continue illustrating PD-L1 blockade immunotherapy and exploring peripheral TCR repertoire in advanced NSCLC patients.

In this study, we confirmed the existence of PD-1^high^ terminal exhausted T cell and PD-1^low^ progenitor exhausted T cell in the enrolled Chinese NSCLC patients, and during treatment, effective anti–PD-L1 immunotherapy was correlated with decreased CD8^+^ PD-1^high^ exhausted T cells. Multiple influencing factors function during the reform of antitumor immunity. Two main subsets constitute the CD8^+^ PD-1–positive T cells: eomes^high^ PD-1^high^ CD8^+^ T cells (terminal T_ex_) respond poorly to the PD-1 pathway blockade; however, T-bet^high^ PD-1^int^ T cells can reverse exhaustion and produce protective immunity *in vivo* (Blackburn et al., 2008). Furthermore, atezolizumab is the inhibitor of both PD-1/PD-L1 and PD-L1/CD80 pathways (Syn et al., 2017), and CD80 on T cells delivered inhibitory signals as a receptor when engaged by PD-L1 (Butte et al., 2007). J. Park et al. demonstrated that by attenuation of the PD-L1/CD80 pathway through treatment with the anti–PD-L1 monoclonal antibody, blockade of PD-L1/CD80 could enhance T-cell expansion and restore response in the previously anergized T cells. So, we hypothesize that anti–PD-L1 antibodies protect the PD-1–positive T cells from apoptosis and T cells renewed from exhaustion without expression of PD-1, which leads to the decrease of CD8^+^ PD-1^high^ T cell during effective atezolizumab treatment.

**FIGURE 5:**
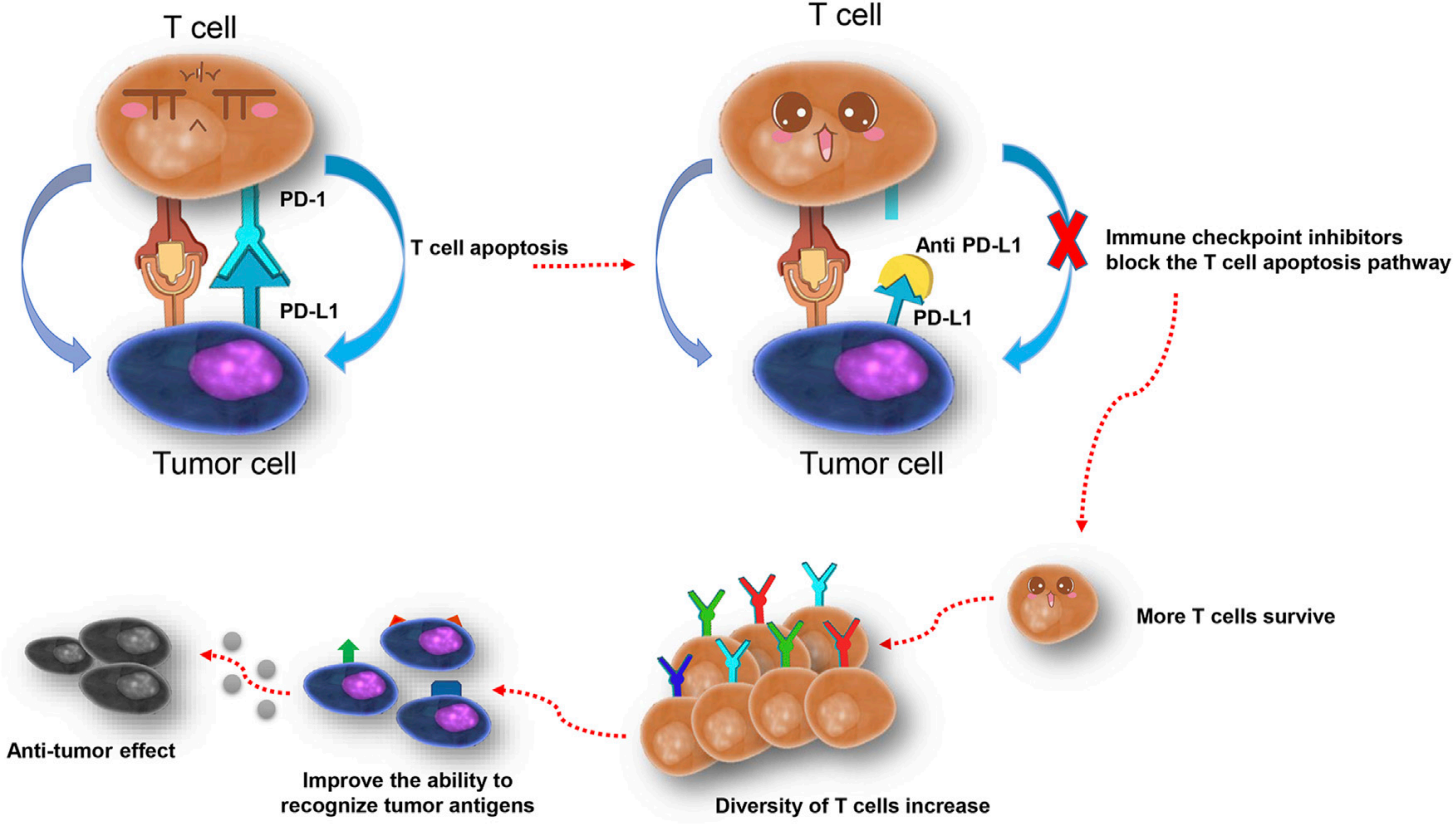
One possible explanation of the increase of T cells and TCR repertoire diversity after PD-L1 blockade.

The blockade of PD-L1 makes more T cells survive from apoptosis, and therefore increases the diversity of T cells and TCR repertoires (Figure 5). Low TCR diversity indicates a severely impaired immune status of patients (Teng et al., 2015; Chen and Mellman, 2017; Snyder et al., 2017), and prospective studies in melanoma patients suggested that peripheral tumor-specific clones are likely to be concentrated in the CD8^+^PD-1^+^ subset (Gros et al., 2016). Therefore, the diversity of the TCR repertoire may reflect the immune status during treatment, and dynamic monitoring could improve its predictive power (Swanton and Govindan, 2016; Jamal-Hanjani et al., 2017). We dynamically analyzed peripheral TCR repertoire diversity in NSCLC patients, increased TCR repertoire diversity, and clonal proliferation that appeared under situations when patients responded to atezolizumab before progression. The TCR remained at a higher level during stable disease but dropped down after progression, which indicated the potential of peripheral TCR repertoire diversity to be a promising monitoring biomarker for checkpoint blockade immunotherapy. Similarly, Wieland et al. (2018) reported oligoclonal expansions in the peripheral CD8^+^ TCR repertoire after pembrolizumab initiation in melanoma patients and identified the activation of tumor-infiltrating CD8^+^ T-cell clones in peripheral blood after anti–PD-1 immunotherapy. In a study containing 29 advanced bladder cancer patients (Snyder et al., 2017), researchers noticed the expansion of circulating tumor-associated TCR 3 weeks after the initiation of atezolizumab, which is correlated with the durable clinical benefit. The further analysis helps us pick two characteristics of the TCR repertoire, the top two clones in CDR3, and singleton frequency (or its percentage), to summarize the changing pattern of the TCR repertoire. No matter the top two clones change or not, the response group always kept a decent or increased percentage of singleton frequency, which increases the possibility of more tumor-specific T cells to control tumor proliferation. Thus, we hypothesize that anti–PD-L1 leads to more tumor antigen presented to T cells, and more tumor-specific T cells survive. Nevertheless, unfortunately, pretreatment singleton frequency of the TCR repertoire could not predict the progression-free survival of NSCLC patients in our study. To further confirm the correlation between TCR repertoire diversity and PFS, a large-scale cohort study is needed.

Targeting cancer immunity deficiency is not a single-factor control experiment; multiple factors and unknown events influence the final results of immunotherapy. Our study noticed that patients who responded to anti–PD-L1 therapy are younger than those who progressed (53.5 vs. 64.8 years, *p* < 0.05, Supplementary Table S2). The impact of age on the therapeutic efficacy of anti–PD-1/PD-L1 treatment is controversial, but phenotypic changes in the adaptive immune system with age certainly include less naïve T cells and more memory T cells, which result in decrease of the TCR repertoire (Nishijima et al., 2016; Daste et al., 2017; Marrone and Forde, 2017).

Several limitations of this study should be noticed. First, all of our patients were from a single institution, and the small cohort restricted the statistical power. So, we focused on describing the features of the TCR repertoire instead of making definite conclusions. Second, considering the patient’s willingness and costs, the information of PD-L1 and other inhibitory receptors’ expression levels were not recorded in every patient; therefore, more detailed analyses were lacking. Third, the percentage of overlap between tumor-infiltrating lymphocytes (TILs) and peripheral TCR repertoire is still in debate, but TILs are not tested. Further studies are needed to confirm whether circulating T cells can represent TILs. Finally, although we defined TCR repertoire changing patterns for early recognition of atezolizumab-sensitive patients, details of standards need large-scaled and well-organized research to confirm.

In conclusion, our study confirmed the existence of T cells with an exhaustion phenotype in Chinese advanced NSCLC patients, and TCR repertoire diversity is correlated with the efficacy of anti–PD-L1 therapy. Moreover, monitoring diversity index or patterns of reshaped TCR repertoires by effective PD-L1 blockade treatment may become the biomarkers during immunotherapy.

## Data Availability

The datasets presented in this study can be found in online repositories. The names of the repository/repositories and accession number(s) can be found in the article/Supplementary Material.
