# Supplementary material for: Deep Sequencing of T-Cell Receptors for Monitoring Peripheral CD8+ T Cells in Chinese Advanced Non–Small-Cell Lung Cancer Patients Treated With the Anti–PD-L1 Antibody
Source: Front Mol Biosci. 2021 Jul 9;8:679130. doi: 10.3389/fmolb.2021.679130 (PMC8299707; doi:10.3389/fmolb.2021.679130)
Supplement: Supplementary file 1 [file DataSheet1.PDF]

Supplementary Materials

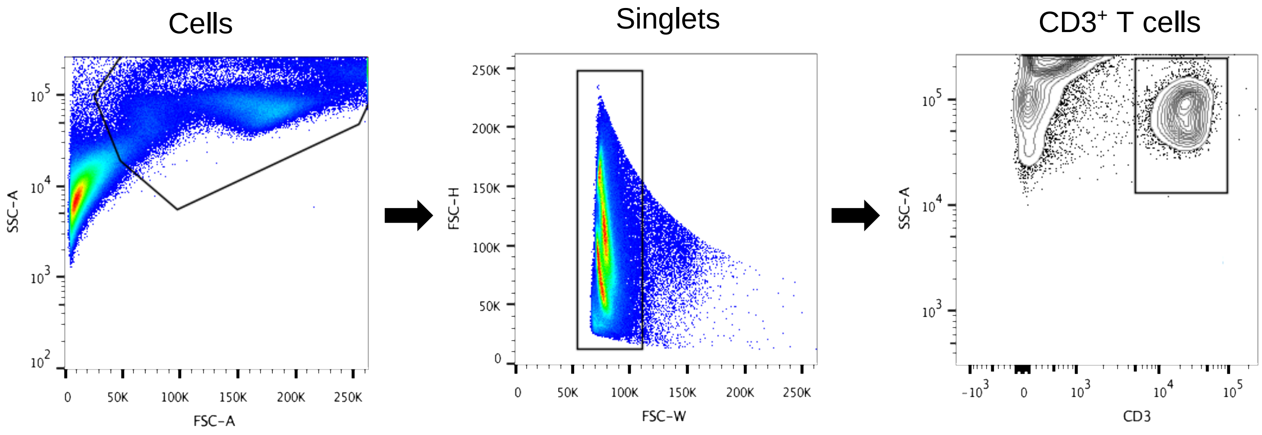

Supplementary Figure 1. The gating strategy for pan T. $CD3^+$ single cells were gated as pan T cells.

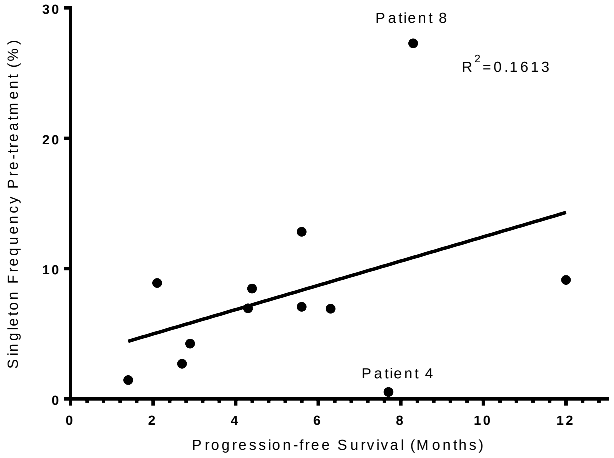

Supplementary Figure 2. The correlation between the frequency of pre-treatment singleton frequency and Progression-Free Survival. (R^2 = 0.16, Linear regression, Graphpad Prism 7)

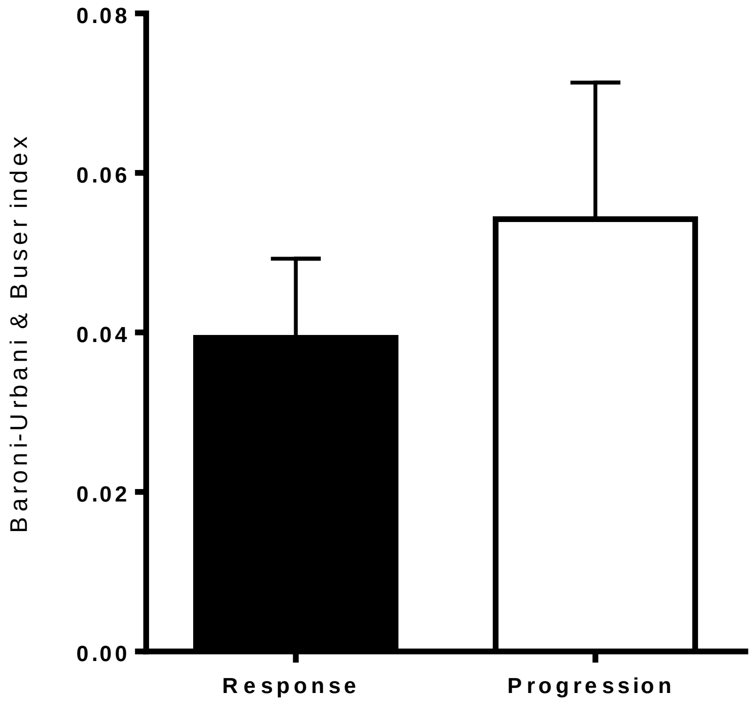

Supplementary Figure 3. The similarity of TCRβ clonotypes before and after treatment calculated by Baroni-Urbani & Buser overlap Index. (the response group vs. the progression group: 0.039 ± 0.0099 vs. 0.054 ± 0.017, $p = 0.44$; unpaired t test)

Supplementary Table 1. The total sequencing reads and successfully-aligned reads

Sample.ID	Total.sequencing.reads	Successfully.aligned.reads
**PT-1pre**	88567	67346
**PT-2pre**	115537	56231
**PT-2post**	602660	473179
**PT-3pre**	125151	50545
**PT-4pre**	81439	45909
**PT-4post**	1280446	991001
**PT-4post2**	69600	68610
**PT-5pre**	235621	233019
**PT-5post**	1236684	833933
**PT-6pre**	246949	245205
**PT-6post**	1271415	957023
**PT-6post2**	1106565	843761
**PT-7pre**	865186	655450
**PT-7post**	1344394	1048205
**PT-8pre**	1449357	1028559
**PT-8post**	1612623	1179440
**PT-9pre**	1443450	1013513
**PT-9post**	1892801	1212496
**PT-9post2**	1573018	1095640
**PT-10pre**	1005541	669947
**PT-10post**	1844883	1385898
**PT-11pre**	439695	436936
**PT-11post**	486962	483170
**PT-12pre**	1576777	1209775
**PT-12post**	352380	349453
**Ave =**	**893908.04**	**665369.8**

The datasets presented in this study can be found in online repositories: https://we.tl/t-o2rsiBrL99.

Supplementary Table 2. Age and gender distribution of the study cohort†

		Anti-PD-L1<br>Response	progression
**age**	**<50**	2	0
	**51-60**	4	2
	**61-70**	0	2
	**>70**	0	2
	**Ave. age (years)***	53.5	64.8
**Gender**	**Female**	2	0
	**Male**	4	6
**Clinical response**	**CR**	0	
	**PR**	1	
	**SD**	5	
	**PD**		6

†: Age and gender distribution of patients in the study cohorts concerning clinical efficacy are shown. *p* values were from the t-test between the response group and the progression group. *: $p < 0.05$

Supplementary Table 3. Singleton frequency analysis of 12 patients.

Response group	Pattern	Donut charts		Top 2 clonotypes		Singleton frequency	
		Before treatment	Before treatment	Before treatment	After treatment	Before treatment	After treatment
**Patient 4**	Pattern 1	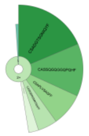	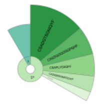	CSADGTSGNIQYF<br>18.617%<br>CASSQGQGGQPQHF<br>13.254%	CSADGTSGNIQYF<br>14.000%<br>CASSQGQGGQPQHF<br>7.159%	1.020%	8.418%
**Patient 6**	Pattern 2	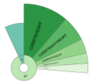	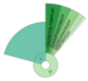	CASSYSYEQYF<br>11.216%<br>CASSFGQGVYNEQFF<br>6.428%	CASRGTGLYNSPLHF<br>4.117%<br>CASSQPGQGTGELFF<br>2.580%	6.924%	20.873%
**Patient 8**	Pattern 2	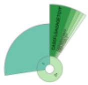	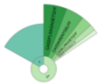	CASSFLGAGAQETQYF<br>5.400%<br>CASSKRENTGELFF<br>1.358%	CASSFLGAGAQETQYF<br>5.891%<br>CASSKRENTGELFF<br>4.565%	27.292%	16.947%
**Patient 9**	Pattern 1	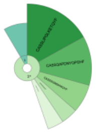	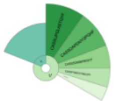	CASSLIPGLKETQYF<br>16.928%<br>CASSQAPDNYQPQHF<br>12.406%	CASSLIPGLKETQYF<br>9.790%<br>CASSQAPDNYQPQHF<br>9.404%	8.465%	19.047%
**Patient 11**	Pattern 2	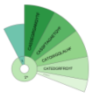	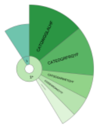	CASSEGRGANGYTF<br>7.791%<br>CASSFTAGAETQYF<br>7.083%	CATQWGQLALHF<br>14.073%<br>CATEDGRFRQYF<br>12.580%	7.597%	9.142%
**Patient 12**	Pattern 1	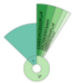	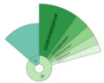	CASSVKGSSGPLHF<br>2.729%<br>CATSSQDNTEAFF<br>2.076%	CASSVKGSSGPLHF<br>9.939%<br>CATSSQDNTEAFF<br>3.714%	12.833%	13.785%

**Progression group**							
**Patient 1**	NA	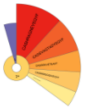	NA	CASHRAGNEYEQYF 11.803% CASSVAGTADYEQYF 8.766%	NA	4.516%	NA
**Patient 2**	pattern 2	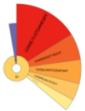	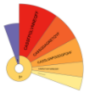	CASSLTLGTGANEQFF 16.121% CASSPNTETYEQYF 6.678%	CASSFPGLVNNEQFF 9.877% CASSQGAGRETQYF 7.241%	2.698%	5.484%
**Patient 3**	NA	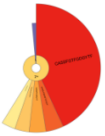	NA	CASSFGTFGDGYTF 43.092% CASSLTSEHRFTDTQYF 4.452%	NA	1.454%	NA
**Patient 5**	pattern 2	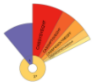	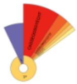	CASSYSYEQYF 7.818% CASSHPTGVEQYF 3.594%	CASSEQGSGYEQYF 9.334% CASSLNKGYGYTF 2.633%	10.925%	7.074%
**Patient 7**	pattern 1	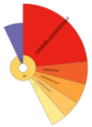	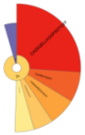	CASSQELLAGGPSDTQYF 23.402% CSARDHYEAFF 5.784%	CASSQELLAGGPSDTQYF 26.107% CSARDHYEAFF 7.593%	6.952%	4.478%
**Patient 10**	Pattern 1	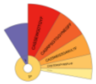	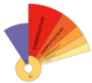	CASSEWGDTQYF 8.453% CASRPSGTGGYNEQFF 7.590%	CASSEWGDTQYF 9.038% CASRPSGTGGYNEQFF 4.571%	8.902%	9.158%
